# Influence of team size on the efficiency of time-critical ALS interventions in prehospital cardiac arrest – A prospective randomised multicentre simulation study

**DOI:** 10.1016/j.resplu.2026.101384

**Published:** 2026-06-10

**Authors:** Sophie Franziska L’habitant, Alina Schenk, Marcus Thudium, Mark Coburn, Alexander Lechleuthner, Florian Piekarski

**Affiliations:** a University of Bonn, University Hospital Bonn, Department of Anesthesiology and Intensive Care Medicine, Germany; b University of Bonn, University Hospital Bonn, Institute for Medical Biometry, Informatics and Epidemiology, Germany; c Cologne University of Applied Sciences, Institute for Rescue Engineering and Civil Protection, Cologne, Germany; d City of Cologne, Firedepartment – Center of Emergency Medicine, Cologne, Germany

**Keywords:** Cardiac arrest, Advanced life support, Emergency medical services, Team size, Simulation study, Prehospital care, Time efficiency

## Abstract

**Objective:**

To determine whether increasing emergency medical service (EMS) team size improves the efficiency of time-critical invasive procedures during advanced life support (ALS) for out-of-hospital cardiac arrest (OHCA).

**Design:**

Randomised prospective multicentre simulation study with a parallel-group comparison of four predefined team sizes.

**Setting:**

Three accredited EMS training centres in North Rhine-Westphalia, Germany.

**Participants:**

210 ALS-trained paramedic students allocated to 59 teams of two to five members.

**Interventions:**

Teams managed a standardised adult ALS scenario (persistent ventricular fibrillation) requiring five invasive procedures: manual defibrillation, supraglottic airway insertion, intravenous access, preparation of amiodarone (300 mg), and preparation of epinephrine (1 mg).

**Measurements and main results:**

The primary endpoint was total scenario time from start to completion of the final procedure. Secondary endpoints were start and completion times for each intervention. Mean total times (mm:ss) decreased progressively with team size: two-person 6:33 ± 0:33 min, three-person 4:13 ± 0:40 min, four-person 2:54 ± 0:24 min, and five-person 2:14 ± 0:19 min (ANOVA, *p* < 0.001). All pairwise differences were statistically significant (Bonferroni-corrected *p*_adj_ < 0.01). Earlier initiation of intravenous access and drug preparation accounted for most time savings. All teams completed every task successfully.

**Conclusions:**

Larger EMS teams performed time-critical ALS procedures significantly faster, with optimal efficiency observed in four- to five-member crews. These findings highlight the operational importance of team composition for prehospital resuscitation performance and may inform staffing policies and simulation-based training.

## Introduction

Out-of-hospital cardiac arrest (OHCA) remains a leading cause of death in Europe. In Germany, approximately 120,000 individuals experience OHCA annually, yet survival to hospital discharge remains below 10%.^1^ Early high-quality cardiopulmonary resuscitation and prompt defibrillation are the strongest predictors of survival, with each minute of delay reducing survival probability by 7–10%.^2^, ^3^, ^4^ Timely administration of epinephrine and amiodarone is similarly associated with higher rates of return of spontaneous circulation.^5^, ^6^, ^7^, ^8^

Beyond clinical algorithms, organisational factors substantially influence resuscitation performance. Emergency medical services (EMS) initiate resuscitation in over 80% of German OHCA cases.^9^ Standard EMS units consist of two professionals, though teams may include three to five members depending on operational demands.^10^ This variability raises a central question: How does team size affect the timely performance of advanced life support (ALS) interventions?

Larger teams can enable parallel task execution, reducing delays inherent to sequential procedures. However, increasing personnel also demands more rigorous communication and coordination. International simulation studies indicate that teams with four or more members perform key ALS interventions, defibrillation, airway management, and medication preparation, more rapidly than smaller teams.^11^, ^12^, ^13^ Evidence from the German EMS system remains limited, which is notable given its structural differences from Anglo-American paramedic-based models. German prehospital care is organised as a physician-based rendezvous system, in which non-physician EMS professionals respond as the first unit on scene and are joined by an emergency physician en route or on arrival, operating under standardised treatment pathways and standard operating procedures. Whether increasing team size improves the efficiency of time-critical ALS procedures within this distinct operational framework has not previously been examined.^14^ The 2025 AHA guidelines update emphasises team size as critical for prehospital resuscitation, recommending that EMS be organised to ensure all essential tasks are performed without time-critical delays.^15^

This prospective multicentre simulation study examines how EMS team size influences the time required to execute invasive ALS procedures in a standardised OHCA scenario. Start times, completion times, and total durations were compared across teams of two to five members. We hypothesised that larger teams would perform time-critical procedures significantly faster than smaller teams. The aim is to provide evidence that can inform staffing strategies and simulation-based training in prehospital resuscitation.

## Methods

### Study design

This prospective, multicentre simulation study was conducted across three EMS training centres in North Rhine-Westphalia, Germany, between September 2024 and February 2025. Participants were randomly allocated to the study groups, ensuring a randomised study design. Paramedics trained in the regional standardised treatment pathways (BPR/SAA NRW 2023) were eligible for inclusion. The study was approved by the Ethics Committee of the University of Bonn (Ref. BO-264/2024), prospectively registered (DRKS00035991), and conducted in accordance with the Declaration of Helsinki. All participants provided written informed consent.

### Scenario and procedures

**Fig. 1:**
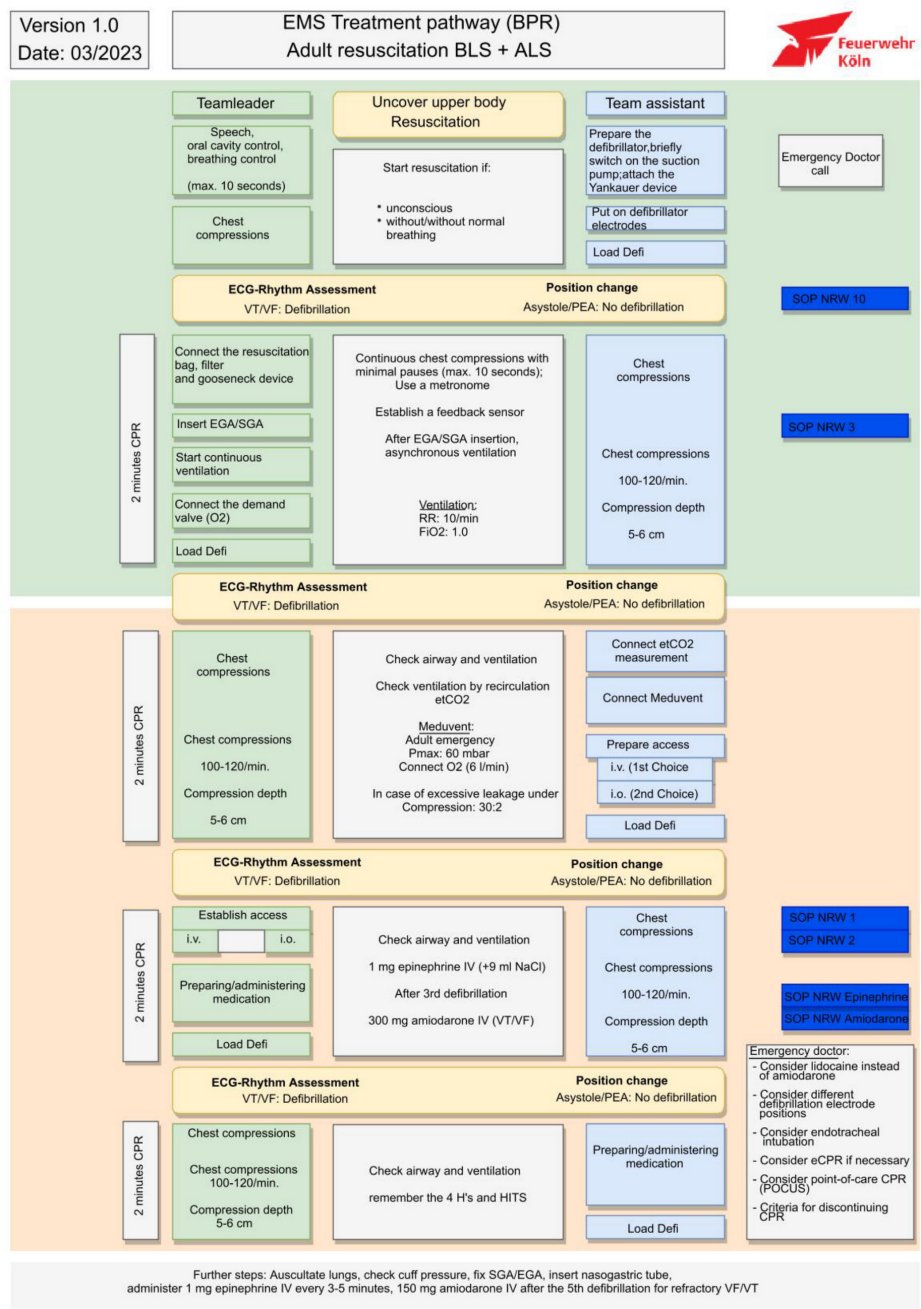
**Resuscitation treatment pathway of Firedepartment Cologne for EMS**.

Teams of two to five members managed a standardised adult ALS scenario featuring persistent ventricular fibrillation on a high-fidelity manikin. The scenario required completion of five predefined procedures according to local SOPs (Fig. 1): manual defibrillation, supraglottic airway insertion, peripheral intravenous access, and preparation of amiodarone and epinephrine. All scenarios were conducted in true real time, with teams adhering strictly to 2-min CPR cycles in accordance with the regional standardised treatment pathways; no time compression was applied.

### Outcome measures

**Fig. 2:**
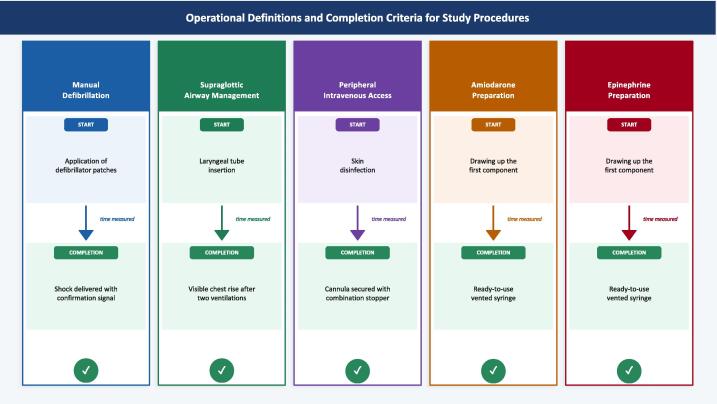
**Start and end points definitions for study procedures in a simulated prehospital resuscitation**.

The primary endpoint was total scenario time, defined as the interval from the audible start signal to successful completion of all five required procedures. Secondary endpoints included the start time, end time, and duration of each individual procedure, procedural success rates, and questionnaire-based data on participants' prior experience, perceived adequacy of team size, and satisfaction with team performance (Fig. 2).

All procedures were operationalized using standardised criteria defined a priori and explained during preparatory training. Manual defibrillation was timed from patch application to shock delivery with confirmation signal; subsequent shocks were assessed qualitatively only. Supraglottic airway insertion began with supraglottic airway device insertion and ended with visible chest rise after two ventilations. Peripheral intravenous access was performed on the manikin’s integrated IV training arm in real time, following the same procedural steps as in clinical practise: skin disinfection, cannula insertion, and securing with a combination stopper. Successful IV access was confirmed by a trained observer per predefined checklist criteria. Preparation of amiodarone and epinephrine was timed from drawing up the first component to a ready-to-use, correctly vented syringe; administration was not included in the time measurement.

Timing was performed by trained observers using digital stopwatches. Start and end times were recorded based on predefined visual triggers and transferred to a standardised measurement table. Times were rounded to the nearest second. In cases of simultaneous events, the first clearly identifiable trigger was recorded. Minimum performance criteria, including minimal pause during defibrillation, visible chest movement, and correct medication volume, were documented using a standardised quality checklist. Scenarios involving protocol deviations, procedural omissions, or duplicate task performance were excluded. No missing data imputation was performed.

### Statistical analysis

Sample size was calculated a priori using G*Power (version 3.1.9.7, Heinrich Heine University Düsseldorf, Germany). Based on one-way ANOVA with four groups, a medium-to-large effect size for the total time, *α* = 0.05, and target power of at least 80%, a minimum of 76 scenarios and 266 participants was required. The target sample was conservatively increased to mitigate potential data loss from technical or procedural issues.

All times are reported as mean ± standard deviation (SD) and 95% confidence intervals where appropriate, in the format mm:ss. Total scenario time, start and end times were compared across the four team sizes using one-way ANOVA (*α* = 0.05). For each comparison, we report the F-test statistic (*F*), associated *p*-value (*p*), and the effect size Omega-squared (*ω*^2^) with 95% confidence interval (CI). Significant omnibus *F*-tests were followed by Bonferroni-adjusted pairwise post-hoc *t*-tests with adjusted *p*-values reported as *p*_adj_ and effect sizes reported as mean differences with 95% CI. Data were screened for outliers prior to analysis. All tests were two-sided, with *p* < 0.05 (*p*_adj_ < 0.05) considered statistically significant. Analyses were performed using SPSS version 26 (IBM Corp., Armonk, NY) and the R language and environment for statistical computing (version 4.5.1.).

## Results

### Participants

**Fig. 3:**
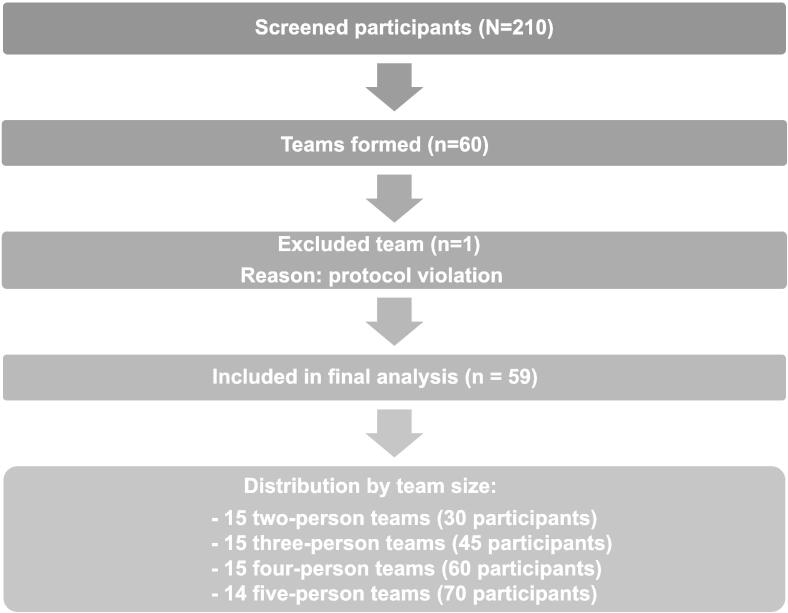
**Flow diagram of study participants with single participation. All participants were independent, and no individual participated more than once in the study**.

The final analysis included 59 team scenarios: 15 each for two-, three-, and four-person teams, and 14 for five-person teams (one excluded due to protocol violation). A total of 210 paramedic trainees participated (Fig. 3). Demographic and professional characteristics were comparable across groups (Table 1).

**Table 1:** Demographic and occupational characteristics of participants by team size.

**Characteristic**	**2-Person** **(*N* = 30)**	**3-Person** **(*N* = 45)**	**4-Person** **(*N* = 60)**	**5-Person** **(*N* = 75)**	**Total** **(*N* = 210)**
Age, years (mean ± SD)	25.9 ± 5.0	28.0 ± 4.6	25.8 ± 4.7	26.6 ± 5.6	26.5 ± 5.1
Sex, male/female, %	76.7/23.3	84.4/15.6	81.7/18.3	77.3/22.6	80/20
Professional experience, years (mean ± SD)	4.0 ± 2.2	3.8 ± 1.9	3.6 ± 3.0	3.4 ± 2.2	3.7 ± 2.4
Prior resuscitation experience, %	100	100	100	100	100
Training year 2nd/3rd, %	57/43	33/67	53/47	66/34	55/45
EMT certification, %	90	96	82	81	86

Values are mean ± SD or *n* (%). No statistically significant differences between groups for any characteristic.

### Primary endpoint

**Fig. 4:**
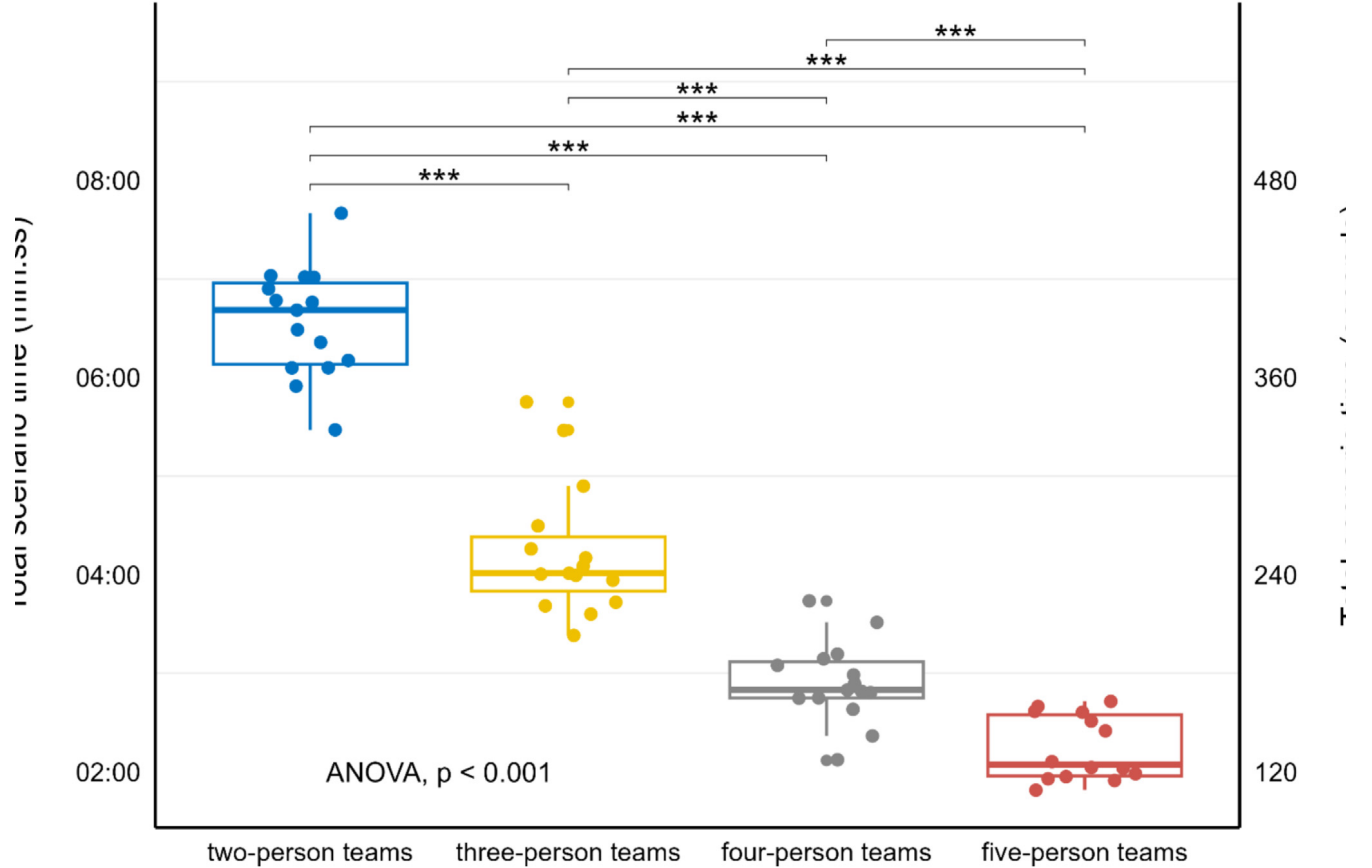
**Total scenario time by team size. Mean times decreased progressively from 6:33 ± 0:33 (two-person teams) to 2:14 ± 0:19 min (five-person teams). One-way ANOVA, *F*(3,55) = 205.54, *p* < 0.001, *ω*^2^ = 0.91 (95% CI: 0.86–0.93) All pairwise comparisons were statistically significant (*p*_adj_ < 0.05)**.

Total scenario time differed significantly across team sizes (ANOVA, *F*(3,55) = 205.54, *p* < 0.001, *ω*^2^ = 0.91 (95% CI: 0.86–0.93)). Mean times decreased progressively with increasing team size: 6:33 ± 0:33 min for two-person teams, 4:13 ± 0:40 min for three-person teams, 2:54 ± 0:24 min for four-person teams, and 2:14 ± 0:19 min for five-person teams. Post-hoc Bonferroni comparisons confirmed significant differences between all pairs, including four- versus five-person teams (mean difference: 0:40 (95% CI: 0:09–1:11) *p*_adj_ = 0.005). These results can be found in Fig. 4.

### Secondary endpoints

Start and end times for all five procedures shifted progressively earlier with increasing team size (Table 2, Fig. 4). The effect was most pronounced for intravenous access and medication preparation, where two-person teams showed substantially delayed initiation compared to larger teams.

**Table 2:** Timing of ALS interventions by team size.

**Intervention**	**Metric**	**2-Person**	**3-Person**	**4-Person**	**5-Person**
Defibrillation	Start	0:22 ± 00:05	0:16 ± 00:04	0:13 ± 00:03	**0:11 ± 00:02**
	End	0:54 ± 00:08	0:51 ± 00:07	0:48 ± 00:10	**0:43 ± 00:09**
	Duration	0:32 ± 00:06	0:35 ± 00:06	0:35 ± 00:07	0:32 ± 00:08

Airway	Start	1:30 ± 00:14	1:08 ± 00:12	0:54 ± 00:14	**0:51 ± 00:12**
	End	2:08 ± 00:10	1:48 ± 00:13	**1:28 ± 00:16**	1:31 ± 00:14
	Duration	0:38 ± 00:09	0:40 ± 00:09	0:34 ± 00:10	0:40 ± 00:11

IV Access	Start	3:30 ± 00:25	1:27 ± 00:35	0:58 ± 00:18	**0:41 ± 00:14**
	End	4:50 ± 00:37	2:59 ± 00:37	2:13 ± 00:24	**1:59 ± 00:26**
	Duration	1:20 ± 00:19	1:32 ± 00:25	1:15 ± 00:14	1:18 ± 00:23

Amiodarone	Start	5:22 ± 00:36	2:57 ± 00:34	1:34 ± 00:24	**0:51 ± 00:15**
	End	5:55 ± 00:35	3:40 ± 00:34	2:12 ± 00:22	**1:25 ± 00:17**
	Duration	0:33 ± 00:14	0:43 ± 00:09	0:38 ± 00:14	0:34 ± 00:11

Epinephrine	Start	6:08 ± 00:31	3:38 ± 00:37	2:17 ± 00:19	**1:18 ± 00:30**
	End	6:33 ± 00:33	4:11 ± 00:41	2:52 ± 00:24	**1:51 ± 00:24**
	Duration	0:25 ± 00:06	0:33 ± 00:14	0:35 ± 00:11	0:33 ± 00:15

Total time	–	**6:33 ± 0:33**	**4:13 ± 0:40**	**2:54 ± 0:24**	**2:14 ± 0:19**

***Note:*** Times reported as mean values (mm:ss). Bold text indicate the shortest (i.e., fastest) time for each metric within each intervention, highlighting the team size that initiated or completed the respective procedure earliest. Duration values are not highlighted, as no significant differences were observed between groups. Total time reported as mean ± SD. Success rate was 100% for all procedures across all team sizes.

*Primary endpoint ANOVA: F (3,55) = 205.54, p < 0.001, ω*^2^ *= 0.91 (95% CI: 0.86 to 0.93). All pairwise comparisons statistically significant (p_adj_ < 0.01).*

**Fig. 5:**
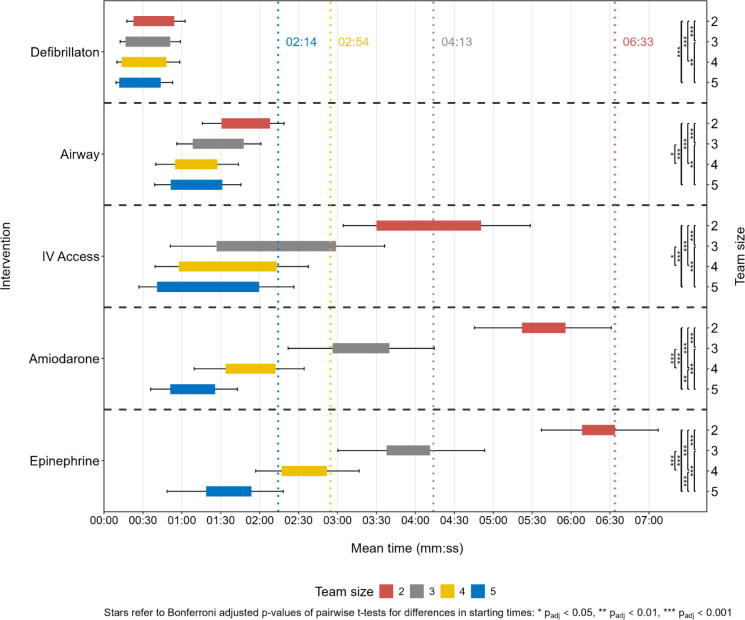
Timeline of ALS interventions by team size. Horizontal bars represent mean start and mean end times for each procedure. Error bars refer to one standard deviation. Dashed vertical lines indicate mean total scenario completion times for each team size. Larger teams initiated all procedures earlier, enabling parallel task execution and substantially reducing total scenario duration. Stars refer to Bonferroni-adjusted *p*-values derived from t-tests performed for each intervention separately (*p_adj_ < 0.05, ^**^p_adj_ < 0.01, ^***^p_adj_ < 0.001).

For defibrillation, mean start times ranged from 0:22 ± 0:05 (two-person) to 0:11 ± 0:02 (five-person teams). Team size showed a statistically significant impact on mean start times (ANOVA, *F*(3,55) = 21.99, *p* < 0.001, *ω*^2^ = 0.52 (95% CI: 0.30–0.63)), with statistically significant differences between two-person teams and all larger groups (Fig. 5). End times followed a similar pattern, though procedure duration remained constant across groups (31–34 s).

Airway management showed statistically significant differences in both start times (ANOVA, *F*(3,55) = 25.20, *p* < 0.001, *ω*^2^ = 0.55 (95% CI: 0.34–0.66)), and end times (ANOVA, *F*(3,55) = 26.75, *p* < 0.001, *ω*^2^ = 0.57 (95% CI: 0.36–0.67)). Post-hoc tests revealed differences between most group comparisons except four- versus five-person teams, suggesting a saturation effect (Fig. 5).

The largest effect sizes were observed for intravenous access (start time: ANOVA, *F*(3,55) = 139.59, *p* < 0.001, *ω*^2^ = 0.88 (95% CI: 0.80–0.91)) and medication preparation. For amiodarone, two-person teams started, on average, at 5:22 ± 0:36 compared to 0:51 ± 0:15 in five-person teams (ANOVA, *F*(3,55) = 246.51, *p* < 0.001, *ω*^2^ = 0.93 (95% CI: 0.88–0.94)), with all pairwise comparisons being statistically significant (Fig. 5). Epinephrine preparation showed similar results (ANOVA, *F*(3,55) = 251.15, *p* < 0.001, *ω*^2^ = 0.93 (95% CI: 0.88–0.95), see Fig. 5).

Procedure durations showed no statistically significant differences across team sizes for any intervention, indicating that time savings resulted from earlier task initiation rather than faster execution (Table 2). All teams achieved 100% procedural success rates.

## Discussion

This simulation study demonstrates that total scenario duration decreased progressively with each additional similarly skilled team member, supporting the hypothesis that larger teams enable parallel task execution and more efficient workflow during prehospital resuscitation. The observed reduction from, on average, 6:33 min in two-person teams to 2:14 min in five-person teams represents a 66% mean decrease in total scenario time, with large effect sizes across most interventions.

The 2025 AHA and ERC guidelines explicitly emphasise the importance of adequately sized teams with clearly defined roles. The AHA recommends that all essential tasks, including chest compressions, airway management, defibrillation, vascular access, medication administration, and team leadership, must be reliably covered.^15^, ^16^, ^17^ The ERC highlights the value of role assignment at the start of each shift and the presence of at least one ALS-trained professional to optimise efficiency in critical interventions.^2^ Our findings provide empirical support for these recommendations by quantifying the time advantages associated with increased team size.

For defibrillation, significant differences in start times were observed between two-person teams and larger groups, as well as between three-person and five-person teams. Smaller teams face competing demands at scenario onset, as patient assessment, equipment preparation, and basic life support measures must be performed simultaneously. This forces a sequential approach and delays shock delivery. Larger teams distribute responsibilities earlier, allowing preparatory measures to occur in parallel while chest compressions continue uninterrupted. Despite the comparatively small number of procedural steps involved in defibrillation, larger teams demonstrated a measurable advantage. This finding aligns with evidence from Herlitz et al. and AHA data identifying early defibrillation as a crucial predictor of survival in both out-of-hospital and in-hospital cardiac arrest, with each minute of delay reducing survival probability by 7–10%.^15^, ^18^ Observational data further confirm that teams with more personnel achieve faster time-to-first-shock and improved survival rates, presumably because resources for rhythm analysis and defibrillator preparation are available sooner.^19^

The influence of team size was particularly evident for airway management, with significant differences in both start and end times across most group comparisons. Notably, four-person and five-person teams showed no significant difference, suggesting a threshold effect at four members beyond which additional personnel provide limited benefit for this intervention. In larger teams, one member can assume responsibility for airway management early in the scenario while others continue chest compressions and prepare for defibrillation. Smaller teams often must prioritise basic measures and defer advanced airway manoeuvres until the most urgent tasks are completed. Tsai et al. similarly reported that larger teams perform advanced airway procedures more efficiently, though their study found optimal performance in six-person teams for endotracheal intubation.^12^ The difference in optimal team size likely reflects the greater technical complexity of intubation compared to the supraglottic airway insertion examined in our study. These findings are consistent with ERC recommendations that resuscitation teams include personnel with advanced airway management competencies.^2^

The largest effect sizes in our study were observed for vascular access (*ω*^2^ = 0.88) and medication preparation (*ω*^2^ = 0.93), indicating that these interventions are most sensitive to team size. The pronounced effect can be explained by the position of these tasks in the resuscitation sequence. In smaller teams, personnel are occupied with chest compressions and airway management during the early phase of resuscitation, preventing simultaneous initiation of vascular access or medication preparation.^20^ This results in substantial delays: two-person teams initiated intravenous access at, on average, 3:30 min compared to 0:41 min in five-person teams. Larger teams can perform multiple tasks in parallel, with dedicated members establishing vascular access and preparing medications while resuscitation continues uninterrupted. This parallel approach significantly reduces time to first medication administration and increases adherence to guideline recommendations, which advise early epinephrine administration in non-shockable rhythms or after the third shock in persistent shockable rhythms.^16^ Tsai et al. reported consistent findings, demonstrating that teams exceeding four members administered epinephrine significantly faster than smaller teams.^12^ Observational data from South Korea provide further support: three-person teams achieved significantly better neurological outcomes than two-person teams (7.2% vs. 5.4%; adjusted OR 1.23), which was attributed in part to earlier vascular access and medication administration enabled by the additional rescuer.^21^

The largest effect sizes were observed for vascular access and medication preparation. However, the clinical interpretation of the medication preparation findings requires contextualisation. In persistent shockable rhythms, epinephrine and amiodarone are recommended after the third defibrillation attempt, typically around four minutes into the resuscitation. In four- and five-person teams, mean scenario times were 2:54 and 2:14 min respectively, meaning that drug preparation in these groups preceded any clinical indication for medication administration. If defibrillation achieves return of spontaneous circulation within the first two cycles, preparation would be unnecessary altogether. In geographic regions where pre-filled syringes are standard, the preparation step as measured here is additionally of limited operational relevance. Medication preparation times should therefore be interpreted as proxies for team workflow organisation and parallel task capacity rather than as direct clinical outcome predictors. The clinically most meaningful findings remain the time advantages in defibrillation, airway management, and intravenous access, where earlier initiation has a direct and evidence-based impact on survival outcomes.

An important observation across all interventions was that procedure duration did not differ significantly between team sizes. This indicates that the time savings observed in larger teams result from earlier task initiation rather than faster task execution. The finding suggests that individual procedural skills were comparable across groups, consistent with the standardised ALS training all participants had received. The advantage of larger teams therefore lies in workflow organisation and the ability to parallelize tasks, not in enhanced technical performance of individual procedures.

These findings also help contextualise the relationship to Martin-Gill et al., who compared two-, three-, and four-person all-paramedic crews in a simulated cardiac arrest and found no significant difference in no-flow fraction or in time to ALS procedures, including intravenous access, medication administration, and endotracheal intubation.^11^ However, the directional trends in their data are consistent with our findings; time to intravenous access, for example, decreased from 259 to 168 s across two- to four-person crews. The authors explicitly acknowledged that their study was underpowered for procedural secondary endpoints, with only 10 scenarios per group and completion rates as low as 50–70% in two-person teams within the fixed 8-min observation window. The present study addressed these limitations through a larger sample, 100% procedure completion across all team sizes, and scenario termination at full task completion rather than a fixed cutoff – likely accounting for why the same directional effect reaches significance here. Of note, Martin-Gill et al. also observed that chest compression quality was compromised in larger teams due to distraction from concurrent ALS tasks, underscoring the importance of structured role assignment, which was systematically operationalised through standardised treatment pathways in the present study.

An important prerequisite for the efficiency gains observed in larger teams is the cross-trained composition of our study teams. All participants were trained to perform each of the five required ALS procedures independently, enabling genuine parallelisation of tasks. This contrasts with multidisciplinary resuscitation teams in which members hold specialised roles, where the parallel execution model may not be applicable. Evidence from neonatal and paediatric resuscitation contexts suggests that in protocols designed for sequential execution with role-specific responsibilities, increasing team size does not yield comparable improvements.^22^, ^23^ Our findings should therefore be interpreted in the context of homogeneous, cross-trained EMS teams and may not generalise to heterogeneous or role-specialised resuscitation teams.

Collectively, our findings indicate that teams of four to five members optimise efficiency for time-critical ALS interventions. The threshold effect observed for airway management and intravenous access, where four-person and five-person teams performed similarly, suggests that four members may represent the minimum team size for efficient parallel task execution in standard ALS scenarios. However, benefits may diminish or reverse when team size exceeds a certain threshold. Excessively large teams risk overcrowding the resuscitation area, communication difficulties, and role confusion, potentially compromising rather than enhancing performance.^24^ Effective team performance therefore requires not only adequate staffing but also clear role assignment, structured communication, and competent leadership to ensure that additional members are utilised efficiently.^25^

### Limitations

Despite the prospective multicentre design, several limitations should be considered. All scenarios were conducted on a high-fidelity manikin in accredited EMS training centres, a setting that differs substantially from real out-of-hospital environments: actual cardiac arrest scenes involve spatial constraints, environmental unpredictability, bystander presence, and significant psychological stressors that cannot be fully replicated under controlled conditions, and may attenuate the observed team size effects in real prehospital practise.

Participants were paramedic trainees rather than experienced professionals, which may limit generalizability to established EMS teams with different skill levels and ingrained team dynamics. However, the use of trainees with standardised baseline competencies ensured that observed differences reflected team processes rather than individual skill deficits.

Blinding of observers was not feasible given the nature of the intervention, potentially introducing measurement bias despite the use of standardised criteria and predefined triggers. Manual timing with digital stopwatches adds inherent variability compared to video-based assessment with independent reviewers.

Additionally, participants recruited from three regional training centres may share institutional characteristics, communication styles, or procedural habits, creating clustering effects not accounted for in the statistical analysis. Future studies might benefit from multilevel modelling approaches to address this hierarchical data structure.

The Hawthorne effect cannot be excluded, as awareness of observation may have enhanced performance beyond typical levels. Finally, the German EMS system, with its specific regulatory framework (NotSanG) and standardised treatment protocols (BPR/SOPs), differs structurally from Anglo-American paramedic-based systems and other international models, limiting direct transferability of findings to other healthcare contexts.

The a priori sample size calculation indicated a requirement of 76 scenarios (266 participants) to detect a medium-to-large effect (Cohen's *f* = 0.35) with 80% power at *α* = 0.05. Due to scheduling constraints and limited availability of training slots during the data collection period, the final sample comprised 59 scenarios (210 participants), representing 78% of the planned sample size. Nevertheless, the observed effect size for the primary endpoint was very large (*ω*^2^ = 0.91, 95% CI: 0.86–0.93; corresponding to a very large Cohen’s *f*), substantially exceeding the originally assumed effect magnitude. Thus, despite the reduced sample, the study retained sufficient statistical power to detect these differences.

## Conclusions

Team size is a key determinant of resuscitation efficiency. In this simulation of prehospital adult VF arrest, increasing team size from two to five members led to progressively faster completion of invasive ALS tasks, with optimal efficiency observed at four to five members. Time savings resulted primarily from earlier task initiation through parallel execution rather than faster procedural performance. These findings support strategies to ensure multi-provider response to OHCA and highlight the importance of effective task distribution in simulation-based training and prehospital resuscitation practise.

## CRediT authorship contribution statement

**Sophie Franziska L’habitant:** Writing – review & editing, Writing – original draft, Methodology, Investigation, Formal analysis, Data curation, Conceptualization. **Alina Schenk:** Writing – review & editing, Methodology, Formal analysis. **Marcus Thudium:** Writing – review & editing. **Mark Coburn:** Writing – review & editing, Supervision. **Alexander Lechleuthner:** Writing – review & editing, Supervision, Resources, Methodology. **Florian Piekarski:** Writing – original draft, Supervision, Methodology, Conceptualization.

## Ethics approval

Ethics approval was obtained from the Ethics Committee of the Medical Faculty of the University of Bonn (reference number: 2024-264-BO).

## Funding

No external funding or financial support was received for this work.

## Declaration of competing interest

The authors declare that they have no known competing financial interests or personal relationships that could have appeared to influence the work reported in this paper.
